# Case of Twins of Extraordinary Size

**Published:** 1848-08

**Authors:** P. G. Bertolet

**Affiliations:** Oley, Pennsylvania


*Case of Twins, of Extraordinary Size.* By P. G. Bertolet, M. D. of Oley, Pennsylvania.

Early on the morning of the 15th of April, 1848, I was requested to meet Dr. Thompson in consultation, at five o'clock, in the following case:

Mrs. A. H., in her sixth labour, was seized with regular pains at 10 o'clock on the previous morning, and at 2 o'clock, after having been in labour four hours, was delivered of a fine, vigorous female child. The vertex presented to the left acetabulum, and the infant, after it was born, weighed nine and a half pounds. The labour pains then ceased for a time, but soon recurred with increased violence. Upon examination, the right hand of another child was discovered, high up in the vagina; this was returned within the uterus, after which the vertex presented very nicely in the second position at the posterior strait. We now had hopes that all further assistance would be unnecessary, but after waiting for a considerable time, the head still continued in exactly the same situation. There had been a constant flow of blood from the time the first child was born, and the patient's strength was becoming exhausted. I succeeded in applying the *long forceps* after some difficulty, and soon delivered the lady of a male child of unusual size; it was still-born, and weighed eleven and a quarter pounds, making an aggregate weight of twenty and three-quarter pounds contained in the uterus beside the secundines.

After the second delivery the hemorrhage ceased, and no unpleasant symptom occurred to retard the quick recovery of the patient. Both mother and child are now perfectly well.

Mrs. H. has had previously to the above, five natural labours and two abortions, immediately preceding her last accouchement. All this took place within twelve years; if she had been safely delivered, at full term, in all these cases, therefore, she would have had nine children.

